# Fascin upregulation in primary head and neck squamous cell carcinoma is associated with lymphatic metastasis

**DOI:** 10.3892/ol.2014.2007

**Published:** 2014-03-28

**Authors:** KONSTANTINOS PAPASPYROU, CHRISTOPH BROCHHAUSEN, IRENE SCHMIDTMANN, KAI FRUTH, HARALAMPOS GOUVERIS, JAMES KIRCKPATRICK, WOLF MANN, JUERGEN BRIEGER

**Affiliations:** 1 Department of Otorhinolaryngology, Head and Neck Surgery, University Medical Center of the Johannes Gutenberg University Mainz, Mainz D-55101, Germany; 2 Institute of Pathology, University Medical Center of the Johannes Gutenberg University Mainz, Mainz D-55101, Germany; 3 Institute of Medical Biostatistics, Epidemiology and Informatics (IMBEI), University Medical Center of the Johannes Gutenberg University Mainz, Mainz D-55101, Germany

**Keywords:** fascin, head and neck squamous cell carcinoma, marker, lymphatic, metastasis

## Abstract

Fascin is an actin-bundling protein that is associated with cellular motility and cancer-cell invasion. The present study aimed to examine the expression of fascin in head and neck squamous cell carcinoma (HNSCC) and its potential use as a biomarker. In a prospective study with a median follow-up time of 48.8 months, tumor tissues, adjacent healthy tissues and cervical lymph node metastases were collected from 25 patients and analyzed by immunohistochemistry. The specimens were scored according to the intensity of fascin staining and the percentage of tumor cells stained using a semi-quantitative scoring approach; the data were analyzed and correlated with clinical follow-up observations. All of the investigators were blinded to the origin of the specimens. The expression levels of fascin were significantly increased in the tumor tissues (P=0.03) and lymph node metastases (P=0.03) compared with that of the normal tissues. The high expression level of fascin in the tumor tissues was correlated with the N-status, however, not with overall survival. Therefore, fascin may be a suitable marker for the prediction of regional lymphatic metastasis in HNSCC.

## Introduction

Head and neck (HN) squamous cell carcinoma (SCC) is the sixth most common type of malignancy worldwide accounting for ~39,000 new cases in the United States annually (1). Early-stage cancer frequently manifests with minimal or no clinical findings and symptoms, resulting in a delayed diagnosis and poor survival rate. Approximately 40% of patients with HNSCC present with early stage disease, and either surgical resection or radiotherapy is recommended as the single treatment modality. The majority of patients (60%) present with a locally advanced disease (2), which requires a multidisciplinary approach, combining surgery, radiotherapy and chemotherapy. Significant additional concerns are second primary tumors (synchronous or later occurring) and distant metastases. Second primary tumors in HNSCC have an incidence rate of 6–20% (3).

The identification of reliable tumor markers for HNSCC is expected to improve the diagnosis and prognosis of patients. Cytogenetic and immunohistochemical analyses have revealed that the overall prognosis in HNSCC patients is correlated with DNA aneuploidy and Ki-67 score (4). Furthermore, human papillomavirus, epidermal growth factor receptor and the mutation status of p53 have been shown to have prognostic value (5). However, currently the only molecular marker that has been established for use in the clinical setting is the SCC antigen (6). Therefore, there is an urgent requirement for novel prognostic markers to guide therapeutic decision-making and improve patient outcome.

The functional role of fascin remains unclear, although experimental data indicates a role in cell motility and the detachment of tumor cells (7). Fascin is involved in the cross-binding of actin bundles to form membrane protrusions and, thus, is significant in cell motility and the migratory changes in carcinogenesis (7). Fascin appears to provide cancer cells with an efficient mechanism to assemble stable long-living invasive protrusions, which allow tumor invasion into the extracellular matrix and disrupt epithelial junctions (8).

Fascin has emerged as an interesting potential biomarker due to its low or absent expression in the majority of normal adult epithelia; colonic (9), breast (10), ovarian (11), stomach (12), pancreas (13), oral cavity (14,15), oropharynx (15), nasopharynx (16) and larynx (17), yet fascin upregulation has been reported in all types of human carcinoma that has been studied to date (17,18). Consistently, primary carcinomas, with high levels of fascin, correlate with a clinically more aggressive disease and poor prognosis (19). However, the number of available studies on fascin expression in upper aerodigestive tract cancers is limited (14–17) and no comparative analysis of the expression in HNSCC-associated macroscopically-normal tissue and HNSCC-metastases has been performed.

In the present prospective study, fascin was analyzed to evaluate its potential as a clinically relevant biomarker in HNSCC.

## Patients and methods

### Patients and tissue specimens

A total of 25 primary tumors collected from 25 adult patients (males, n=22; females, n=3) with histologically confirmed HNSCC were included in this explorative prospective study. The patients underwent surgery between 2004 and 2009 at the Department of Otorhinolaryngology, in the Head and Neck Surgery of a tertiary referral center (University Medical Center of the Johannes Gutenberg University Mainz, Mainz, Germany). The exclusion criteria were as follows: Recurrent HNSCC tumors at the same site; second primary tumors in the presence of previous HNSCC in neighboring sites; and cases of *in situ* carcinoma. The median follow-up time was 48.8 months. Regarding the tumor samples, a particular specimen slide was selected based on whether the transition area (invasion front), between the tumor and healthy tumor-adjacent epithelial tissues, could be observed, however, the two could be clearly differentiated from each other.

The present study was reviewed and approved by the Institutional Review Board of the University Medical Center of Johannes Gutenberg University Mainz (Mainz, Germany) and performed in accordance with the Declaration of Helsinki. Written informed consent was obtained from all of the patients.

### Immunohistochemistry

**Figure 1:**
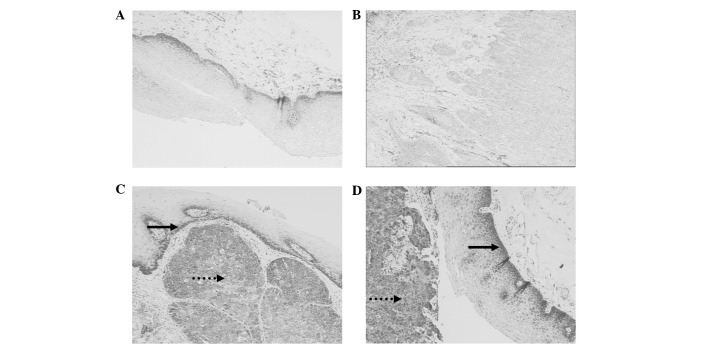
Immunohistochemical analysis of fascin expression (magnification, ×100). (A) Hodgkin lymphoma tissue served as the positive control revealing an integrated score: 2 (moderate positive stain, only basal cells stained). (B) Integrated score: 3 (moderate positive stain, 50–80% of tumor cells stained) in the cervical lymph node metastasis. (C) Integrated score: 5 (moderate-strong positive stain, >81% of tumor cells stained) in the tumor tissue (disrupted arrow); and 2 (moderate positive stain, only basal cells stained) in the healthy epithelial tissue (continuous arrow). (D) Integrated score: 6 (strong positive stain, >81% of tumor cells stained) in the tumor tissue (dashed arrow); and 4 (moderate-strong positive stain, basal epithelial zone stained) in the healthy epithelial tissue (continuous arrow).

Immunohistochemical analysis of formalin-fixed, paraffin-embedded specimens was performed using standard procedures (20). Heat-induced antigen retrieval was performed, using microwave treatment (3×5 min each; 600 W in 10 mM citrate buffer, pH 6.0), on all of the slides following dewaxing and rehydration; blocking of endogenous peroxidase with 3% H_2_O_2_ methanol was subsequently performed. Following pre-incubation with 10% normal serum in 2% bovine serum albumin (BSA)/phosphate-buffered saline (PBS) for 20 min (to avoid unspecific binding), primary antibodies were incubated overnight at 4°C. A monoclonal antibody raised in mice against the epitope fascin (1:50; Dako Deutschland GmbH, Hamburg, Germany) was used. The slides were incubated with a biotinylated secondary antibody (1:100; DAKO Deutschland GmbH), streptavidin peroxidase (1:100; Dianova GmbH, Hamburg, Germany) and 3,3′-diaminobenzidine/H_2_O_2_ (1.85 mM). All of the washing procedures were performed in PBS and dilutions of antibodies were prepared in 2% BSA/PBS at room temperature. The slides were counterstained with hematoxylin and eosin. The primary antibody was substituted with PBS and served as the negative control, and Hodgkin lymphoma tissue was used for positive control staining (Fig. 1A). The staining reaction was quantified using a scoring system that was modified according to Bittinger *et al* (21). Immunostained specimens were independently examined by two investigators and, in the instance of a discrepancy, by a third individual; the investigators were blinded to the origin of the specimen. Briefly, specimens were scored according to the intensity of staining (0, none to weak; 1, weak; 2, moderate; 3, strong) and the percentage of tumor cells stained (0, 0–24% positive; 1, 25–50% positive; 2, 51–80% positive; 3, >81% positive), or the cell layers of the healthy epithelial tissue specimens that were stained (0, negative; 1, only basal cells; 2, all the cells beyond superficial cells; 3, all the cells). The scores for the intensity of staining and the percentage of stained cells were summated to yield an integrated staining score. The four groups were compared for statistical analysis. Specimens with an integrated score of 0, 1–2, 3–4 and 5–6 were included in groups 0, 1, 2 and 3, respectively.

### Statistical analysis

The association between categorical variables was analyzed using contingency tables and Fisher’s exact tests (two-sided). P≤0.05 was considered to indicate a statistically significant difference and the P-values were considered to be descriptive as they were not adjusted for multiplicity. Survival (overall and event-free) is described by Kaplan-Meier estimates and the statistical analysis was performed using SAS 9.2 (SAS Institute Inc., Cary, NC, USA) and SPSS 15.0 (SPSS Inc., Chicago, IL, USA).

The present study aimed to determine whether fascin expression in tumors or healthy epithelia is a predictor of survival by investigating overall, relapse-free and event-free survival. In the first case, only mortalities were considered to be events, in the second case only relapses were considered and in the third case, mortalities, relapses and second primary tumors were considered to be events. In addition, gender, pathological (p) tumor-node-metastasis (TNM), tumor site, smoking, alcohol, chemotherapy, radiation therapy and tumor grade were examined as further potential explanatory variables. The possible predictors were assessed separately by computing Kaplan-Meier estimates for each stratum and compared survival with strata using the log-rank test.

In order to assess whether one type of tissue exhibited systematically higher fascin expression compared with another, a sign test was performed comparing tumor tissue, cervical lymph node metastases and healthy tissue.

## Results

### Clinical data

In total, tissue samples from 25 patients were analyzed, including tumor samples from 23 patients, healthy epithelial tissue samples from 20 patients and cervical lymph node metastases from eight patients. The median age was 62 years (range, 39–77 years). Tobacco consumption and alcohol abuse history were positive in 20 and 17 patients, respectively. The patients with IDs 1, 6, 5 and 13 had a HNSCC of the paranasal sinuses, larynx, hypopharynx and oropharynx, respectively. Only one of the 25 patients developed a second primary tumor and four patients experienced recurrence (Table I).

### Immunohistochemical analysis of fascin expression and survival

**Figure 2:**
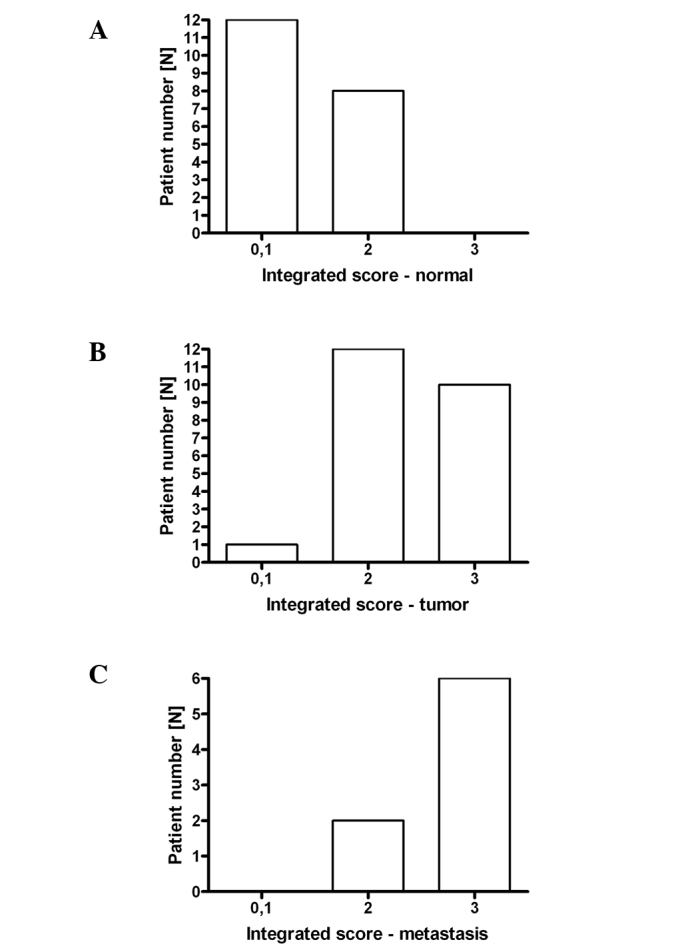
Fascin expression levels. The integrated score groups in (A) healthy epithelial tissue, (B) tumor tissue and (C) metastasis. Groups, by integrated score: 0; 1 (1–2); 2 (3–4); and 3 (5–6). Fascin expression levels were significantly increased (P≤0.05) in the tumor and metastases tissues compared with the normal tissue.

The specimens from healthy tumor-adjacent epithelial tissue, tumor tissues and cervical lymph node metastases tested positive for fascin. Increased fascin expression levels were found in tumor tissue and cervical lymph node metastases samples when compared with the expression levels in the healthy epithelial tissue. From the 20 available healthy epithelial tissue specimens, 12 had an integrated score of 1–2 (group 1) and eight of 3–4 (group 2; Fig. 2A). From the 23 available tumor specimens, one had an integrated score of 1–2 (group 1), 12 of 3–4 (group 2) and 10 of 5–6 (group 3; Fig. 2B). Among the eight available cervical lymph node metastases, two (one of which is depicted in Fig. 1B) had an integrated score of 3–4 (group 2) and six of 5–6 (group 3; Fig. 2C). The expression levels of fascin were significantly increased in the tumor tissue (P=0.03) and lymph node regional metastasis (P=0.03) compared with the normal tissue, as detected via the sign test, while there was no systematic difference (P=1.00) when comparing between the tumor tissue and lymph node metastases. In addition, increased fascin expression was observed at the invasion front of the tumor in all of the samples (Fig. 1C and D).

**Figure 3:**
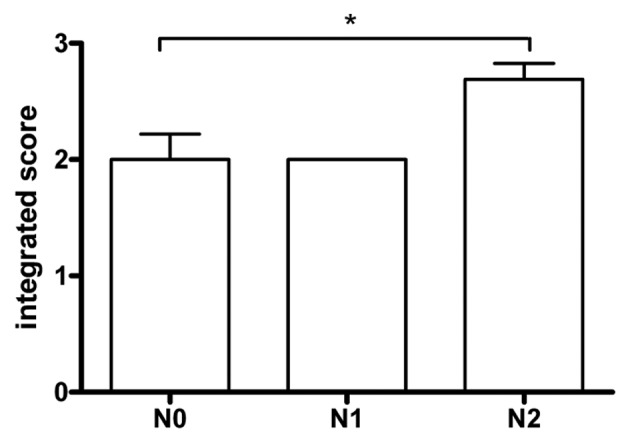
Fascin expression significantly correlated with the pathological node stage of the tumor tissues (^*^P=0.05).

The fascin expression levels in the tumor tissues were not associated with pT (P=0.56) or pM stage (P=0.63), smoking status (P=1.00), tumor grade (P=1.00), alcohol consumption (P=0.18), gender (P=0.53) or tumor localization (P=0.07), however, an association (P=0.05) was observed between fascin expression in the tumor tissues and pN stage (Fig. 3). The association between fascin expression in lymph nodes and other factors was not investigated due to the low sample number.

The fascin expression levels in the healthy epithelial tissue were not associated with pT (P=0.90), pN (P=1.00) or pM (P=1.00) stage, smoking status (P=0.62), tumor grade (P=1.00), alcohol consumption (P=0.64), gender (P=0.40) or tumor localization (P=0.85).

**Figure 4:**
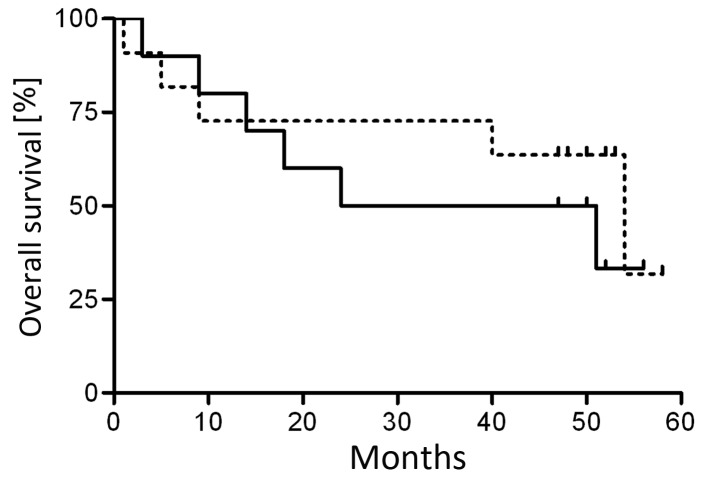
Fascin expression and overall survival were not identified to be significantly correlated (P=0.38). Fascin high group (continuous line; integrated-score group 3) vs. fascin low group (dotted line; integrated-score groups 0, 1 and 2).

For overall survival, the univariate analyses did not support the hypothesis that fascin expression, in tumor and healthy epithelial tissues, is a predictor for overall, relapse-free and event-free survival (Table II). To further analyze the affect of fascin expression on overall survival, the tumor samples were grouped as fascin high and fascin low. The high group included tumor samples with fascin integrated scores of 5–6 (group 3) and the low group included those with integrated scores of 0–4 (groups 0–2). Despite a median overall survival of only 38 months in the fascin high group, compared with 54 months in the fascin low group, no significant correlation was observed between fascin expression and overall survival (Fig. 4 and Table II).

## Discussion

The present study investigated fascin expression in primary HNSCC, the tumor invasion front, surrounding healthy tissues and lymph node metastases. A longer median overall survival in the group with low fascin expression levels was observed, however, was not identified to be statistically significant, which is in accordance with previous studies associating fascin expression with survival. Wu *et al* (16) reported that in nasopharyngeal SCC, the overall survival and disease-free survival rate for patients with high fascin expression was significantly lower compared with patients exhibiting low fascin expression. Lee *et al* (14) reported that the mean overall survival of patients with oral SCC and high tumor fascin expression was 34 months compared with 49 months in patients with no or low fascin expression levels.

A notable observation in the present study was that fascin expression in tumors is correlated with pN status, which is in accordance with the decreased overall survival of patients with high fascin-expressing primary tumors. Furthermore, Vignjevic *et al* (22) found an increased fascin level in primary colon cancer tissues was associated with clinical distant metastases. Fascin participates in the regulation of cell adhesion, interactions and migration and is an F-actin interacting protein, which forms parallel actin bundles that are found in leading edge protrusions of mesenchymal cells (23). In epithelial cells, *de novo* expression of fascin induces protrusions and increases motility (24). Previous *in vitro* studies demonstrated that elevated levels of fascin increased the speed of cell migration and emphasized the association between fascin overexpression and the motility of transformed cells in urothelial carcinoma (8). Disruption of endogenous fascin expression in nasopharyngeal carcinoma cells using the small interfering RNA technique suppressed nasopharyngeal carcinoma cell invasiveness, and decreased cell filopodia and lamellipodia, thus, indicating the relevance of fascin to cancer cell invasiveness (16). These data may therefore indicate the possible role of fascin in the pathogenesis of lymphatic metastases.

Fascin expression has been found to be low or absent in the majority of normal adult epithelia of varying origin (9). However, increased fascin expression in the basal layer of nasopharyngeal epithelial tissues has previously been reported (16), which supports our observation of frequent and increased fascin expression in healthy, although tumor-adjacent, epithelial tissue. The observed upregulated fascin expression may reflect a tissue-specific expression pattern or an association between fascin and the proliferating capacity of cells. Alternative explanations may be that the epithelial tissue that was examined was directly adjacent to the tumor and the tumor may condition its microenvironment, or that the macroscopically-normal epithelial tissue was not normal at a molecular level. The latter is supported by our previous study regarding genetic alterations similar to those found in the primary tumors in the tumor-adjacent normal tissue (3,25). Previous studies have found increased levels of fascin in dysplastic epithelia (26) or fascin levels increasing gradually in the progression from normal epithelia to simple hyperplasia, dysplasia, carcinoma *in situ*, to invasive esophageal SCC (26), supporting the assumption that unexpected observation of fascin expression in normal epithelia may reflect pre-malignant changes at the molecular level.

The findings of the present study support the hypothesis that fascin is involved in HNSCC. A possible mechanism may be the increased motility of fascin-expressing cancer cells. As a consequence, patients with high tumor fascin levels may be at a higher risk for a more aggressive tumor and therefore, should be treated accordingly, i.e. fascin expression may have relevance for therapeutic decision-making. For example, in patients with questionable symptoms who may undergo neck lymph node surgery, such as a marginal case when ultrasonography is not sufficient to determine metastasis from enlarged cervical lymph nodes, a patient exhibiting high fascin levels in the tumor tissues would be classified as high risk and may receive surgery, while a patient with low fascin levels would not. However, such an approach requires further investigation.

In conclusion, the present study provides evidence of the role of fascin in HNSCC metastasis. Thus, fascin should be evaluated further as a potential molecular marker for the prediction of regional lymphatic metastasis in HNSCC.

## Figures and Tables

**Table I:** Clinical and histological data of patients.

				Integrated score groups 0–3			
					
ID	Age at first surgery (years)	Gender	pTNM	Fascin (tumor)	Fascin (healthy epithelium)	Fascin (cervical lymph node metastasis)	Recurrence
1	52	F	T2N1M0	n.a.	n.a.	3	No
2	50	M	T1N2M0	3	1	n.a.	Yes
3	58	M	T1N2M1	3	n.a.	2	No
4	63	F	T3N1M1	2	2	3	No
5	39	F	T2N2M0	2	n.a.	n.a.	No
6	65	M	T1N2M0	3	1	n.a.	No
7	73	M	T3N0M0	3	1	n.a.	No
8	52	M	T3N2M0	2	2	n.a.	Yes
9	62	M	T4N2M0	3	n.a.	n.a.	No
10	53	M	T3N0M0	2	2	n.a.	No
11	61	M	T1N0M0	2	2	n.a.	Yes
12	65	M	T2N2M0	3	2	3	No
13	63	M	T1N1M0	2	1	3	No
14	68	M	T1N2M0	3	1	3	No
15	77	M	T4N0M0	2	n.a.	n.a.	No
16	49	M	T2N2M0	3	1	2	No
17	76	M	T1N2M0	2	2	3	No
18	69	M	T4N2M1	3	1	n.a.	No
19	65	M	T2N2M0	2	2	n.a.	No
20	45	M	T2N1M0	2	1	n.a.	No
21	45	M	T2N0M0	2	1	n.a.	No
22	53	M	T2N0M0	2	1	n.a.	Yes
23	46	M	T2N0M0	1	2	n.a.	No
24	64	M	T3N0M0	n.a.	1	n.a.	No
25	71	M	T1N2M0	3	1	n.a.	No

0, integrated score of 0; 1, integrated score of 1–2; 2, integrated score of 3–4; 3, integrated score of 5–6; n.a., not available; pTNM, pathological tumor-node-metastasis; M, male; F, female.

**Table II:** P-values obtained from log-rank tests for possible predictors of survival.

Explanatory variable (integrated score group)	Overall survival	Relapse-free survival	Event-free survival
Fascin (1 vs. 2 vs. 3)	0.58	0.74	0.71
Fascin (0–2 vs. 3)	0.38	0.78	0.78
Healthy epithelium (1 vs. 2)	0.42	0.94	0.94

0, integrated score of 0; 1, integrated score of 1–2; 2, integrated score of 3–4; 3, integrated score of 5–6. P<0.05 was considered to indicate a statistically significant difference.
